# The Medical Kipling—Syphilis, Tabes Dorsalis, and Romberg's Test

**DOI:** 10.3201/eid1006.AD1006

**Published:** 2004-06

**Authors:** Setu K. Vora, Robert W. Lyons

**Affiliations:** * New York Weill Cornell Medical Center, New York City, New York, USA;; † St. Francis Hospital and Medical Research Center, Hartford, Connecticut, USA

**Keywords:** syphilis, tabes dorsalis, ataxia, neurologic examination, medicine in literature, history of medicine, Rudyard Kipling

Born of expatriate parents in Bombay, India, in 1865, Rudyard Kipling was the first English author to win the Nobel Prize for literature. He received this honor when he was not yet 42 years old. Indeed, Kipling's career is remarkable for its precocious success. His collection of verse Departmental Ditties was published when he was 20 years old. When he first went to England in 1889, he was already a well-known writer.

In his short story Love-o'-Women, published in 1893 in the collection Many Inventions, Kipling gives a clinically accurate description of tabes dorsalis and what is probably the only literary description of Romberg's test. Love-o'-Women was published when Kipling was 27, a year after he had married Caroline Balestier, an American woman, and moved to Dummerston, Vermont. Kipling remained in Vermont for 4 years, during which time he wrote The Jungle Book, The Second Jungle Book, Captains Courageous, and The Seven Seas.

## Kipling and the Doctors

Kipling's life and works are full of references to medicine, but they have received little attention in medical history, as pointed out by W.K. Beatty in a definitive article (*1*) published in 1975. Beatty gives credit to E.F. Scarlett for drawing attention to Kipling's "medical writings" in his article The Medical Jackdaw—Kipling and the Doctors (*2*). Kipling's interest in medicine is evident from one of his earliest verses, "The Song of the Sufferer," which he wrote after a bout of fever and sore throat at age 13. The verse concludes, "For the doctor has harrowed his being,/ And of medicine wondrous the might is;/ He suffers in agony, seeing/ He is prey to acute tonsilitis."

As a schoolboy, Kipling acquired a copy of Culpeper's Herbals—a book by the 17th-century English physician Nicholas Culpeper. Many years later, in 1909, Kipling would write the story A Doctor of Medicine, based on Culpeper's life story. In 1910, he sent his friend Sir William Osler (Regius Professor of Medicine at Oxford) a copy of Rewards and Fairies with a letter thanking Osler for inspiring the story. Another story, Marklake Witches, which features Rene Laennec, the inventor of the stethoscope, also drew on the life of William Osler.

Kipling had many other physician friends. He acknowledged the help of his personal physician, James Conland, whom he called "the best friend I made in New England," for helping him create an authentic American setting for his adventure story Captains Courageous. Kipling's most famous poem, "If," was a tribute to yet another physician friend, Leander Starr Jameson. Jameson was involved in the Boer Wars in South Africa and eventually became prime minister of the Cape colony.

Sir John Bland-Sutton, Kipling's close friend and physician for many years in England, was associated with Middlesex Hospital. In October 1908, probably through the good offices of Bland-Sutton, Kipling gave the introductory lecture to students starting their preclinical studies at Middlesex Hospital. The picture of Kipling and Bland-Sutton in top hats on their way to the hospital for this lecture was the frontispiece of Bland-Sutton's autobiography, The Story of a Surgeon. He dedicated the book to Kipling with the inscription "To my old friend Rud. Critic and adviser." At the annual meeting of the Royal College of Surgeons in 1923, Bland-Sutton gave the Hunterian Oration, and Kipling gave the banquet speech, entitled Surgeons and the Soul. Kipling's fascination with medicine from childhood, exposure to various diseases during his stay on the Indian subcontinent, and close association with many physicians and hospitals provided rich medical material for his stories.

## Medicine and Love-o'-Women

This story, which like many of Kipling's early works is set within another story, takes place in India. Kipling, in the guise of newspaper reporter, attends the murder trial of a sergeant and aggrieved husband, who killed an adulterous soldier. Terrance Mulvaney, another soldier to whom the murder recalls similar events, relates the main story to Kipling, who records it in dialect (*3*).

Mulvaney relates the story of his fellow soldier and friend, Larry Tighe, with whom he served in the Black Tyrone Regiment. Tighe was a strong and handsome man who had a reputation as womanizer. He was also a "gentleman-ranker," a term in the British army for men of the upper class who served as enlisted men, usually because of some disgrace. Mulvaney loses touch with Tighe for many years until he meets him again on a battlefront on India's northwestern frontier. Mulvaney describes the meeting as follows: "‘Larry,' sez I, ‘how is ut wid you?' ‘Ye're callin' the wrong man,' he sez, wed his gentleman's smile, ‘Larry has been dead these three years. They call him Love-o'-Women now'…"

Mulvaney notices later that Tighe staggered a little and leaned over all twisted when he got up off the ground. He also finds Tighe to be suicidal, trying to draw fire from the tribal enemies. Tighe would not go see a doctor and was very careful to hide his disorder. One day while walking around the camp, he stopped and struck his right foot on to the ground three or four times, unable to feel it. Mulvaney notices the military doctor, Lowndes, observe Tighe's actions and confront him. "‘Hould on there,' sez the doctor; an' Love-o'-Women's face, that was lined like a gridiron, turns red as brick. ‘Tention,' sez the docthor; an' Love-o'-Women stud so. ‘Now shut your eyes,' sez the doctor. ‘No, ye must not hould by your comrade.' ‘Tis all up,' sez Love-o'-Women, thrying to smile. ‘I'd fall, doctor, an' you know ut.'"

The doctor ordered Tighe to the hospital, and Mulvaney is dumbstruck to see Tighe crippling and crumbling at every step. He walked with his hands on Mulvaney's shoulder "all slued sideways, an' his right leg swingin' like a lame camel." His hands went all ways at once, and he could not button his tunic. Later, Mulvaney asks the doctor what ails his friend. "‘They call ut Locomotus attacks us,' he sez, ‘bekaze,' sez he, ‘ut attacks us like a locomotive, if ye know fwhat that manes. An' ut comes,' sez he, lookin' at me, ‘ut comes from bein' called Love-o'-Women.'"

This story, written around 1893, accurately describes the clinical syndrome of tabes dorsalis—popularly known as locomotor ataxia at that time. Larry Tighe managed to hide his affliction in spite the agonizing pain. Kipling identifies the social stigma and shame attached to certain neurologic diseases and sexually transmitted diseases. He also shows the private suffering, guilt, and depression prevalent in patients with chronic, disabling, painful disorders. Tighe was driven to suicide attempts from his disease. He also tried to self-medicate with alcohol to make the pain and disability bearable. His secret was given away when he stomped the ground with his foot and did not feel it. Dr. Lowndes caught him in this act and made an excellent instant diagnosis. What follows is a textbook demonstration of Romberg's test, in which a patient with peripheral ataxia experiences increased clumsiness and gait disturbances when asked to close his eyes. The diagnosis of "Locomotus attacks us" seemed well known, even to Tighe, who whispered it to the doctor. Lowndes gave a euphemistic explanation about causality of locomotor ataxia to Mulvaney by telling him that it came from being called "Love-o'-Women." Kipling seems aware of the association between sexual promiscuity, syphilis, and locomotor ataxia.

The story concludes with Tighe's death in a brothel in the arms of his wife, whom he had ruined. His wife, still holding his body, killed herself with a pistol. The doctor proclaimed their deaths attributable to "naturil causes, most naturil causes." The two were buried together with Anglican rites in the civil cemetery "an' the docthor—he ran away wid Major—Major Van Dyce's lady that year—he saw to ut all."

## Kipling's Sources

Nitrini elegantly summarized the history of tabes dorsalis (*4*). In 1836, English physician Marshall Hall described a patient with loss of postural control in darkness caused by severely compromised proprioception. He did not develop the idea further. Moritz Heinrich Romberg is widely credited for the first description of tabes dorsalis around 1840. He listed excessive drinking and sexual activity among the possible causes. Inspired by Hall, he devised the eponymous bedside test. In 1858, Guillaume Duchenne wrote an almost complete clinical description of tabes dorsalis, which he named progressive locomotor ataxia, and hinted at the possibility of syphilis as the cause. It was only in 1875 that Jean-Alfred Fournier firmly advanced syphilis as the cause of tabes dorsalis. The English neurologist Sir William R. Gowers supported the causal relationship between syphilis and tabes and published the data in the Lancet in 1881 (*5*). In his classic textbook, A Manual of Diseases of the Nervous System, published in 1888, Gowers gave accurate details of the modern Romberg's test. Kipling was evidently well acquainted with Gowers and, at a banquet in 1898, stated that he was proud to associate with "real fighting men of his class" (*1*). Interestingly, even Osler gave an excellent clinical description of the clinical features of tabes and Romberg's test in the first edition of his textbook, The Principles and Practice of Medicine (*6*). This textbook was published in 1892, a year before Love-o'-Women. Very likely Kipling became aware of the association between syphilis and locomotor ataxia through Gowers or Osler.

If we take Gowers's publication in the Lancet as acceptance of the causal association between syphilis and tabes in the English medical literature, it took Kipling less than 12 years to incorporate this relatively new syndrome into his work. The disorder is interesting from a literary perspective because it is caused by past sexual indiscretion and almost has a karmic quality about it. Unlike tuberculosis, which was very prevalent at that time, or other major epidemics that gripped the world, locomotor ataxia was not well-known enough to be made the center of a story. It was probably the combination of his natural interest in medical matters and his close association with experts on tabes dorsalis that led Kipling to write Love-o'-Women.

## Diseases in Fiction

In our era of telecommunication and rapid dissemination of information, Ebola hemorrhagic fever, recognized in 1976, took only 12 years to be fictionalized: in 1988, the medical thriller Outbreak by Robin Cook made Ebola a popularly dreaded name. AIDS took only 4 years from initial publicity in 1981 to make its literary and artistic debut in the play, The Normal Heart, by Larry Kramer. Both Ebola and AIDS have tremendous psychologic impact on a global scale, besides their obvious lethality. Extensive media coverage has made these diseases household names and inspired many literary and creative works. Kipling's achievement seems especially brilliant as he was operating in the 19th century and still managed to keep his creations contemporary and to present a relatively obscure disorder in a humane way for a general audience. To our knowledge, Love-o'-Women is one of the earliest examples of literary description of a newly understood medical disorder and a diagnostic test associated with it.
